# Molecular mechanism of monodisperse colloidal tin-doped indium oxide nanocrystals by a hot-injection approach

**DOI:** 10.1186/1556-276X-8-153

**Published:** 2013-04-02

**Authors:** Yizheng Jin, Qing Yi, Yuping Ren, Xin Wang, Zhizhen Ye

**Affiliations:** 1 State Key Laboratory of Silicon Materials, Department of Materials Science and Engineering, Zhejiang University, Hangzhou, 310027, People's Republic of China; 2 Cyrus Tang Center for Sensor Materials and Applications, Zhejiang University Hangzhou, Zhejiang, 310027, People's Republic of China; 3 College of Chemistry & Materials Engineering, Wenzhou University, Wenzhou, Zhejiang Province, 325027, People's Republic of China

**Keywords:** ITO, Molecular mechanism, Synthesis, FTIR, Nanocrystals

## Abstract

Molecular mechanisms and precursor conversion pathways associated with the reactions that generate colloidal nanocrystals are crucial for the development of rational synthetic protocols. In this study, Fourier transform infrared spectroscopy technique was employed to explore the molecular mechanism associated with the formation of tin-doped indium oxide (ITO) nanocrystals. We found that the reaction pathways of the indium precursor were not consistent with simple ligand replacements proposed in the literature. The resulting understanding inspired us to design a hot-injection approach to separate the ligand replacements of indium acetate and the aminolysis processes, generating quality ITO nanocrystals with decent size distributions. The hot-injection approach was readily applied to the synthesis of ITO nanocrystals with a broad range of tin doping. Structural, chemical, and optical analyses revealed effective doping of Sn^4+^ ions into the host lattices, leading to characteristic and tunable near-infrared surface plasmon resonance peaks. The size control of ITO nanocrystals by multiple hot-injections of metal precursors was also demonstrated.

## Background

Colloidal nanocrystals are an important class of functional materials for both fundamental studies and practical applications due to their remarkable properties and excellent solution processability [1-3]. Research on synthetic chemistry of colloidal nanocrystals paves the way to the development of a wide range of potential applications. In the past 2 decades, enormous efforts have been devoted to explore the crystallization kinetics and mechanisms of high-quality colloidal nanocrystals, focusing on the size and shape evolution [4-12]. However, knowledge on the chemical reactions, especially the molecular mechanisms of precursors associated with the formation of colloidal nanocrystals is still limited. For example, the Alivisatos group suggested that for CdSe nanocrystals, precursor conversion limited the rate of nanocrystal nucleation and growth. Size control of the CdSe nanocrystals could be achieved by tuning the reactivity of precursor molecules [13]. Ozin et al. found that the sulfur-alkylamine solution, a widely used ‘black box’ precursor for sulfur, *in-situ* generated H_2_S upon heating, which reacted with metal salts to form metal sulfide nanocrystals [14]. Peng and co-workers demonstrated that the rate-limiting step for synthesis of CdS nanocrystals was the reduction of elemental sulfur by 1-octadecene (ODE), which possessed a critical temperature of *ca.* 180°C [15]. These reports demonstrate that understanding on molecular mechanisms of the chemical reactions is crucial for the development of rational synthetic protocols for colloidal nanocrystals.

Transparent conducting oxides (TCOs) are degenerately doped semiconductor oxides that possess attractive combination of electrical conductivity and transparency to visible light. ITO is the most widely used TCO because of its superior performance in terms of optical transparency and electrical conductivity as well as its excellent chemical and environmental stability. Nowadays, ITO is applied for many applications, such as transparent electrodes for displays, light-emitting diodes or solar cells, and infrared reflector for energy-saving windows [16-20].

The synthesis of colloidal ITO nanoparticles has attracted considerable research interest. This is largely motivated by the goal of employing low-temperature and cost-effective solution processable techniques to deposit ITO thin films on flexible substrates [21]. Early attempts to obtain ITO nanoparticles by the co-precipitation approach in aqueous media generally led to nanoparticles with broad size distribution and poor colloidal stability [22,23]. Niederberger and co-workers suggested that the nonaqueous route involving solvothermal treatments of metal precursors in benzyl alcohol may result in relatively uniform crystalline ITO nanoparticles [24]. A few recent studies demonstrated that quality colloidal ITO nanocrystals could be obtained by nonaqueous approaches [25-30]. It is noteworthy that in 2009, Masayuki and co-workers reported the synthesis of ITO nanocrystals with tunable surface plasmon resonance (SPR) peaks by controlling the concentrations of tin doping [28]. This finding is the first example of tunable SPR in the near-infrared (NIR) region for oxide nanoparticles. The strong SPR in the NIR region of ITO nanocrystals arising from the presence of high concentrations of free carriers was confirmed by Radovanic and co-workers [30]. In a recent publication, the Milliron group further suggested that the localized surface plasmons of ITO nanocrystal films could be dynamically controlled by electrochemical modulation of the electron concentrations, which is promising for future development of energy-saving coating on smart windows [31].

Here we provide a detailed study on the synthesis and characterization of quality monodisperse colloidal ITO nanocrystals with characteristic and tunable SPR peaks in the NIR region. The molecular mechanism of the synthetic method developed by Masayuki et al., which will be called as the Masayuki method in the following text for the sake of presentation, was probed using the Fourier transform infrared spectroscopy (FTIR) technique. The resulting understanding inspired us to modify the synthetic procedures and design a hot-injection approach to synthesize ITO nanoparticles. The key features of the ITO nanocrystals from the hot-injection approach including valance states of tin dopants and molar extinction coefficient were identified. We further applied the hot-injection approach to the synthesis of ITO nanocrystals with a broad range of tin dopants and developed multiple injection procedures, aiming to achieve size control of the products.

## Methods

### Material

Indium acetate and tin(II) 2-ethylhexanoate were purchased from Sigma-Adrich (St. Louis, MO, USA). ODE, *n*-octylether, and oleylamine were purchased from Acros Organics (Fair Lawn, NJ, USA). Tetrachloroethylene (C_2_Cl_4_) and 2-ethylhexanoic acid were purchased from Alfa Aesar (Ward Hill, MA, USA). Hydrochloric acid (HCl), ethyl acetate, and *n*-hexane were analytical grade reagents from Sinopharm Chemical Reagent Co., Ltd. (Shanghai, China). All chemicals were used without further purification.

### The Masayuki method

We repeated the synthesis of ITO nanocrystals using the Masayuki method according to the previous report [28]. Note that the carboxylic acid in the starting materials was changed from *n*-octanoic acid, which was used in the literature [28], to 2-ethylhexanoic acid according to Dr. Masayuki Kanehara's kind suggestions because the use of *n*-octanoic acid led to the formation of ITO nanoflowers, instead of nanoparticles, with significantly broadened SPR peaks (Additional file 1: Figure S1). The proportion of the tin precursor in the reagents, i.e., [tin(II) 2-ethylhexanoate] / ([tin(II) 2-ethylhexanoate] + [indium acetate]), was set to be 10 mol.% because this dopant ratio generated ITO nanocrystals with relatively high free electron density and strong SPR in the NIR region [28]. In a typical reaction, indium acetate (1.08 mmol), tin(II) 2-ethylhexanoate (0.12 mmol), 2-ethylhexanoic acid (3.6 mmol), oleylamine (10 mmol), and ODE (10 ml) were loaded in a three-neck flask and stirred at 80°C under vacuum for 30 min to obtain a clear solution. The solution was heated at 150°C for 60 min under an argon atmosphere. The reaction temperature was further raised to 280°C and stabilized for 2 h to generate ITO nanocrystals. The ITO nanocrystals were precipitated out by adding ethyl acetate, purified, and redispersed in C_2_Cl_4_.

### The hot-injection approach

In a typical reaction, indium acetate (1.08 mmol), tin(II) 2-ethylhexanoate (0.12 mmol), 2-ethylhexanoic acid (3.6 mmol), and ODE (10 ml) were loaded in a three-neck flask and stirred at 80°C under vacuum for 30 min. The solution was heated at 150°C under an argon atmosphere for 60 min before raising the temperature to 290°C. A separate solution of ODE (5 ml) containing oleylamine (10 mmol) at 220°C was rapidly injected into the reaction flask. The reaction mixture was then kept at 290°C for 2 h to obtain ITO nanocrystals.

### Fourier transform infrared spectroscopy analysis

FTIR spectra were recorded on a Bruker Tensor 27 FTIR spectrophotometer at room temperature (Bruker AXS, Inc., Winooski, VT, USA). The samples were prepared by directly spotting hot aliquots onto CaF_2_ plates. Note that in many spectra shown in the paper, we used very thick films to maximize the absorption signals, which may cause saturation of intensities of some relatively strong peaks.

### Powder X-ray diffraction analysis

X-ray diffraction (XRD) measurements were performed on an X'Pert PRO system (PANalytical, Almelo, The Netherlands) operated at 40 keV and 40 mA with Cu KR radiation (*λ* = 1.5406 Å).

### Transmission electron microscopy analysis

Transmission electron microscopy (TEM) images were recorded using a JEOL JEM 1230 microscope (JEOL Ltd., Akishima-shi, Japan) operated at 80 keV. High-resolution TEM (HRTEM) was performed on a Tecnai G2 F20 S-TWIN microscope (FEI, Hillsboro, OR, USA) operated at 200 keV.

### X-ray photoelectron spectroscopy analysis

X-ray photoelectron spectroscopy (XPS) were recorded on a Thermo ESCALAB-250 spectrometer (Thermo Fisher Scientific, Waltham, MA, USA) using a monochromatic Al Kα radiation source (1,486.6 eV).

### Ultraviolet-visible near-infrared absorption spectra analysis

Ultraviolet-visible near-infrared absorption (UV-vis-NIR) spectra of the samples were recorded on a UV 3600 UV-vis-NIR spectrophotometer (Shimadzu, Kyoto, Japan).

### Inductively coupled plasma atomic emission spectroscopy analysis

The purified ITO nanocrystal samples were dissolved in concentrated HCl solutions (36% to 38%). The metal ions were transferred to aqueous phase by extraction twice with distilled water. Elemental analyses were carried out using an IRIS Intrepid II XSP inductively coupled plasma atomic emission spectroscopy (ICP-AES) equipment (Thermo Fisher Scientific, Waltham, MA, USA).

## Results and discussion

FTIR is a powerful tool for the identification of the molecular mechanism associated with the formation of the oxide nanocrystals [7,11,32-34]. For instance, Peng and co-workers found that in the reaction system, to obtain In_2_O_3_ nanocrystals, hydrolysis and alcoholysis were the major reaction pathways for the indium precursors [33]. In a recent study, we showed that the aminolysis approach accounted for the formation of tin-doped ZnO nanocrystals [11].

**Figure 1:**
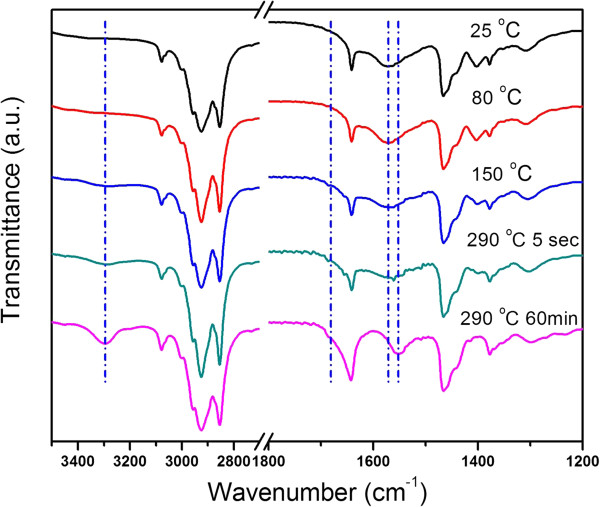
Temporal evolution of the FTIR spectra of the Masayuki method.

We prepared ITO nanocrystals following the Masayuki method and monitored the reactions by recording the FTIR spectra of the aliquots withdrawn from the reaction flasks at different stages, as shown in Figure 1. At a first glance, the molecular mechanism associated with the formation of the ITO nanocrystals is identified as amide elimination through aminolysis of metal carboxylate salts which generates secondary amides, as indicated by the characteristic vibrations at 3,300 (*ν*_N-H_), 1,684 (shoulder, amide I band, *ν*_C=O_), and 1,550 cm^−1^ (amide II band, in-plane *δ*_N-H_) in the FTIR spectra of the solutions which were reacted for 1 h (bottom curve, Figure 1) [35].

Rational choice and design of the metal precursors is one of the most critical issues that control the chemical kinetics of the amide elimination reactions. In the Masayuki method, indium acetate and tin(II) 2-ethylhexanate were used as the initial metal precursors. It was proposed that the acetate groups of indium precursor may be replaced by the long-chain carboxyl groups by introducing free carboxylic acid, i.e., 2-ethylhexanate acid and stirring the reaction mixture of the metal precursors, 2-ethylhexanate acid, oleylamine, and the solvent, at 80°C under vacuum [28]. Nevertheless, we found that the reaction pathways of indium acetate, the initial indium precursor, were debatable because this hypothesis was not consistent with the following facts. As shown in Figure 1, no characteristic peaks of carboxyl acid were observed in the FTIR spectrum of the reaction mixtures at room temperature (top curve). The FTIR spectra of the reaction mixtures exhibited no significant changes after stirring the reaction mixtures at 80°C under vacuum. In addition, the origin of the peak at 1,573 cm^−1^ in the room temperature FTIR spectra of the reaction mixtures is worthy of further discussion since this peak is not consistent with any characteristic peaks of the reagents, i.e., oleylamine, indium acetate, tin(II) 2-ethylhexanate, 2-ethylhexanatic acid, and ODE (Additional file 1: Figure S2).

**Figure 2:**
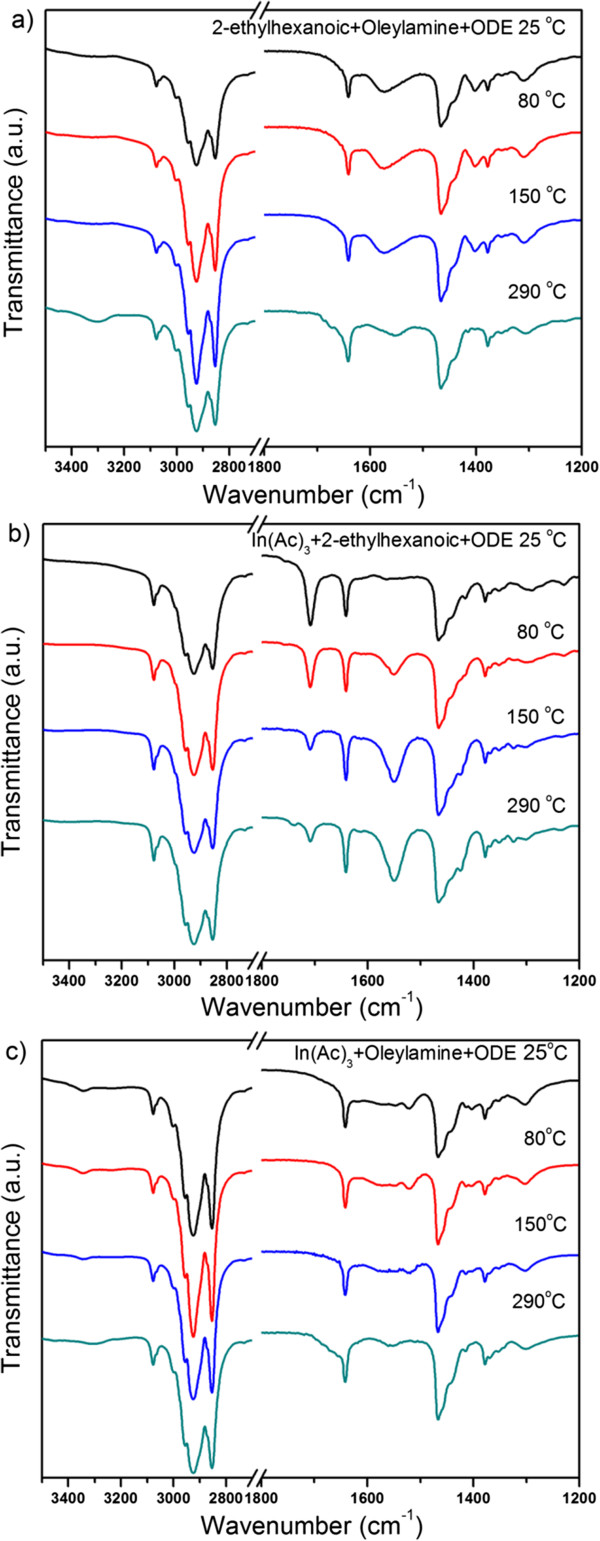
**FTIR spectra.** Of (**a**) 2-ethylhexanatic acid (3.6 mmol) and oleylamine (10 mmol) in ODE, (**b**) indium acetate (1.2 mmol) and 2-ethylhexanatic acid (3.6 mmol) in ODE, and (**c**) indium acetate (1.2 mmol) and oleylamine (10 mmol) in ODE.

We conducted three sets of controlled experiments to gain more insights on the pathways of the indium acetate by recording the temperature-dependent FTIR spectra (Figure 2) of the mixtures of 2-ethylhexanatic acid (3.6 mmol) and oleylamine (10 mmol) in ODE, indium acetate (1.2 mmol) and 2-ethylhexanatic acid (3.6 mmol) in ODE, and indium acetate (1.2 mmol) and oleylamine (10 mmol) in ODE, respectively. Figure 2a showed that 2-ethylhexanatic acid reacted with oleylamine at room temperature, as implied by the absence of the characteristic peak of carboxylic acid at 1,708 cm^−1^ (*ν*_C=O_). This acid-base reaction was a reversible process which gave an ammonium carboxylate salt [36], leading to the peak at 1,573 cm^−1^ in the FTIR spectra. FTIR data also suggested that further heating the ammonium carboxylate salt to 290°C drove off water and resulted in the formation of amide (Figure 2a). Regarding the mixture of indium acetate and 2-ethylhexanatic acid in ODE, we observed that indium acetate was insoluble at room temperature. Raising the temperature to 80°C initiated the replacements of the acetate groups by 2-ethylhexanate. The ligand replacement did not go to completion even when the temperature of the system was as high as 290°C, as revealed by the remaining peak of 2-ethylhexanatic acid at 1,708 cm^−1^ in the FTIR spectra (Figure 2b, bottom). Therefore, the resulting soluble indium compound was carboxylate salts with mixed ligands. Quantitative analyses on the FTIR spectra (Additional file 1: Figure S3) [37] suggested that the ratio of 2-ethylhexanate to acetate was about 3. For the mixture of indium acetate and oleylamine in ODE, the entire reaction system became a clear solution at 80°C. The dissolution of indium acetate by forming complex with oleylamine led to a broad peak between 1,620 and 1,540 cm^−1^ in the FTIR spectra (Figure 2c). FTIR data further revealed that the aminolysis of indium acetate took place when the reaction temperature reached 290°C.

Based on the above facts, we suggest that the reaction pathways of the indium acetate in the Masayuki method is more complicated than simple ligand replacement by 2-ethylhexanate. The peaks at 1,573 cm^−1^ that were observed in FTIR spectra of the reaction mixtures at room temperature, 80°C or 150°C (Figure 1) were due to the formation of ammonium carboxylate salts which consumed free 2-ethylhexanatic acid. The dissolution of indium acetate at 80°C was because of the formation of oleylamine-indium acetate complex, instead of ligand replacement by free carboxylic acid. Given the condition that oleylamine was excessive in the reaction systems, a plausible deduction was that the oleylamine-indium acetate complex was responsible for the formation of ITO nanocrystals. We tested this hypothesis by conducting controlled experiments in which 2-ethylhexanate acid was absent in the reagents. No nanocrystals but agglomerations with poor colloidal stability were formed, implying an exorbitantly fast reaction kinetics of the oleylamine-indium acetate complex. Therefore, the presence of 2-ethylhexanate acid in the starting materials was critical to obtain high-quality ITO nanocrystals for the Masayuki method. This was also reflected by the fact that ITO flowers, instead of nanoparticles, formed when *n*-octanoic acid, instead of 2-ethylhexanate acid, was used in the starting materials (Additional file 1: Figure S1). We suspect that although majority of the 2-ethylhexanate acid reacted with oleylamine to form ammonium carboxylate salts, considering the reversible nature of the acid-base reaction, 2-ethylhexanate acid may impact in the formation of the oleylamine-indium carboxylate complex with adequate reaction kinetics. Nevertheless, such a process is complicated. Modifications on the Masayuki method that induce evident evolutions of the metal precursors are desirable.

**Figure 3:**
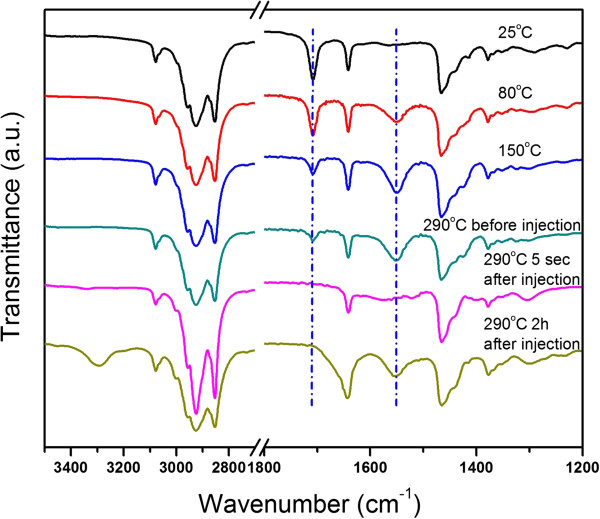
Temporal evolution of the FTIR spectra of the hot-injection approach.

In this regard, we designed a hot-injection approach, which separated the ligand replacements of the indium acetate and the aminolysis reactions of the metal precursors. Indium acetate was reacted with 2-ethylhexanate acid at 150°C for 1 h, allowing sufficient conversion of the indium precursor. Then, the injection of the oleylamine at 290°C initiated the aminolysis processes to obtain ITO nanocrystals. Temporal evolution of FTIR analyses (Figure 3) on the reaction mixtures from the injection approach demonstrated the validity of our proposed reaction pathways of ligand replacements.

**Figure 4:**
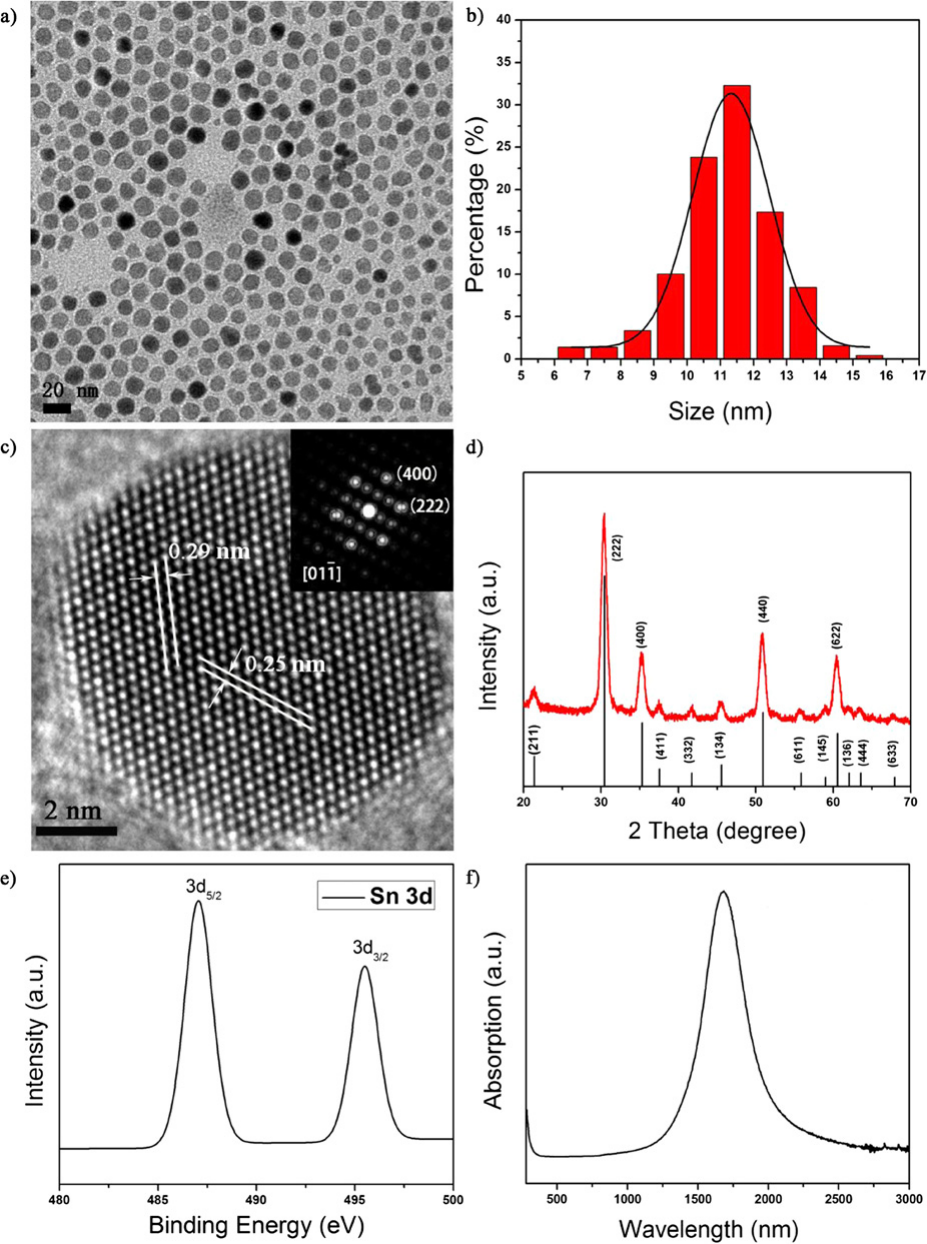
**ITO nanocrystals (10 mol.% of tin precursor) from the hot-injection approach.** (**a** and **b**) A typical TEM image and the corresponding histogram of size distribution of the ITO nanocrystals. (**c**) A typical HRTEM image and the corresponding FFT patterns. (**d**) XRD pattern, (**e**) XPS narrow scan spectrum of the Sn 3d peaks, and (**f**) UV-vis-NIR spectrum.

The synthesis of ITO nanocrystals starting with 10 mol.% of tin precursor in the reagents were used as an example for the products obtained by the hot-injection approach. We conducted a time-dependent study of the particle morphological formation [38,39]. The corresponding TEM images (Additional file 1: Figure S4) revealed the generation of small crystals at 3 min after the injection of oleylamine. The small particles gradually developed into nanocrystals with decent size distributions. The final product after 2 h of reaction had an average diameter of 11.4 ± 1.1 nm (Figure 4a,b). The monodisperity of ITO nanocrystals from the hot-injection approach is moderately improved compared with that of the ITO nanocrystals obtained using the Masayuki method (Additional file 1: Figure S5). HRTEM analyses reveal the high crystalline nature of the ITO nanocrystals. As shown in Figure 4c, the well-resolved lattice fringes with interplanar spacing of 0.29 and 0.25 nm correspond to the {222} and {400} lattice planes of the corundum-type In_2_O_3_, respectively. No nanocrystals that have crystal structures similar to that of SnO or SnO_2_ were found in the HRTEM observation, in line with the electron diffraction analyses (Additional file 1: Figure S6). These results are supported by the XRD characterizations (Figure 4d) that the diffraction pattern matches well with the structure of the corundum-type In_2_O_3_ (JCPDS: 06-0416). ICP-AES analyses on the aqueous solution coming from digestion of the ITO nanocrystals suggest a doping concentration ([Sn] / ([Sn] + [In])) of 9.9 mol.%.

The valence state of tin dopants is critical in terms of modifying the electronic properties of the ITO nanocrystals. Note that aminolysis of pure tin(II) 2-ethylhexanoate, the tin precursor used in our experiments, by oleylamine may lead to tin(II) oxide or tin(IV) oxide depending on specific reaction conditions, as demonstrated by our controlled experiments (Additional file 1: Figure S7). XPS was employed to identify the chemical states of the tin dopants. As shown in Figure 4e and Additional file 1: Figure S8, the binding energy of Sn 3d_5/2_ peak locates at 487.1 eV, which corresponds to the Sn^4+^ bonding state [40,41]. The incorporation of Sn^4+^ ions into the lattice of the nanocrystals led to high free electron concentrations, as confirmed by the characteristic near-infrared SPR peak (Figure 4f). We determined the extinction coefficient per molar of ITO nanocrystals at the SPR peak of 1,680 nm to be 4.5 × 10^7^ M^−1^ cm^−1^, by assuming that the nanocrystals are spherical and 11.4 nm in diameter.

**Figure 5:**
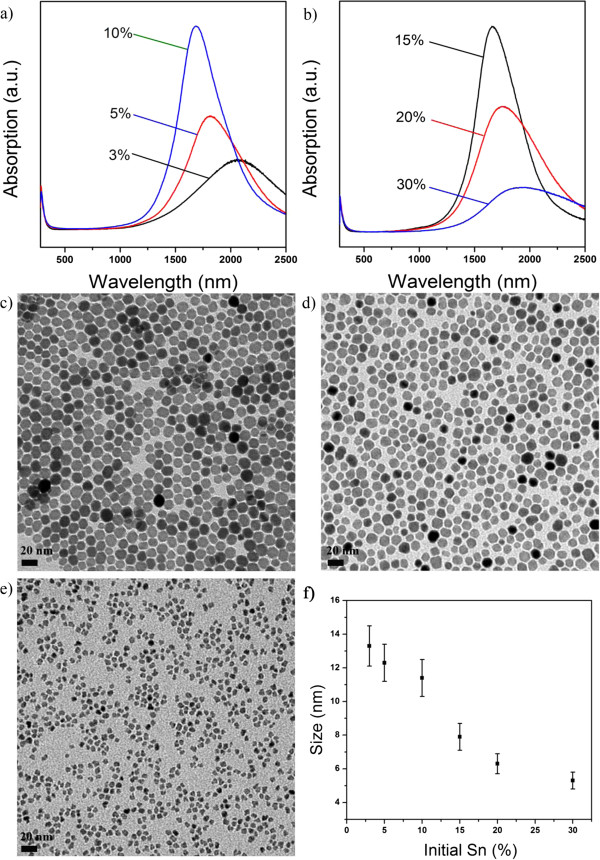
**ITO nanocrystals from the hot-injection approach.** (**a** and **b**) UV-vis-NIR spectra of ITO nanocrystals starting with different molar ratios of tin precursors. (**c**, **d,** and **e**) Typical TEM images of ITO nanocrystals starting with 3, 5, and 30 mol.% of tin precursors, respectively. (**f**) The corresponding size distribution of ITO nanocrystals.

The hot-injection approach is readily applied to the syntheses of ITO nanocrystals with a broad range of tin dopants. As shown in Figure 5a,b, the SPR peak of the ITO nanocrystals gradually blueshifted from 2,100 to 1,680 nm when the ratio of the dopant precursor increased from 3 to 10 mol.%. Further increasing the ratio of the dopant precursor to 30 mol.% resulted in the red shift of the SPR peak to 1,930 nm. The evolution of SPR peaks of ITO nanocrystals from the hot-injection approach is in agreement with that of the ITO nanocrystals from the Masayuki method. TEM observations (Figure 5c,d,e,f) indicated that the sizes of the ITO nanocrystals became smaller, and the standard derivation was kept as ≤10% when high concentrations of tin dopants were used. Nevertheless, when the Sn amount exceeded 15%, the shape of ITO nanocrystals became irregular (Figure 5e). We suggest that this phenomenon is due to the high concentration of tin dopants in the host indium oxide lattices which causes significant lattice distortion.

**Figure 6:**
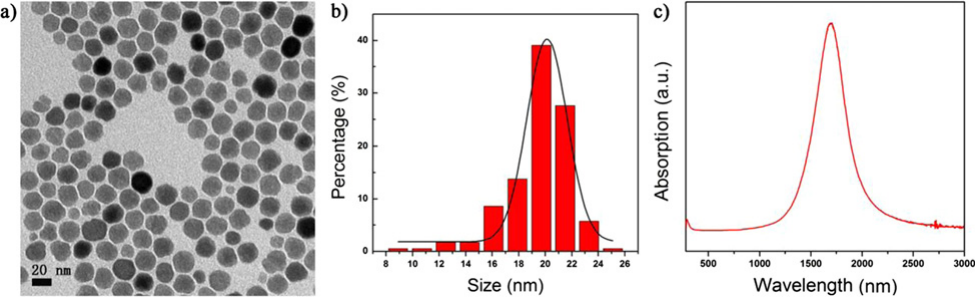
**ITO nanocrystals obtained by multiple injections of reagents.** (**a** and **b**) A typical TEM image and the corresponding histogram of size distribution. (**c**) UV-vis-NIR spectrum.

We further propose effective size tuning of monodisperse ITO nanocrystals via multiple injections of reagents into the reaction mixtures. For example, the diameters of the ITO nanocrystals starting with 10 mol.% of tin precursor were increased from 11.4 ± 1.1 to 20.1 ± 1.5 nm (Figure 6a,b) using the multiple injection approach. The NIR SPR features of the ITO nanocrystals with large diameters were preserved after the multiple injection procedure, as shown in Figure 6c.

## Conclusions

In conclusion, we provide a detailed study on the synthesis and characterization of monodisperse colloidal ITO nanocrystals. The molecular mechanism associated with the formation of the ITO nanocrystals was identified as amide elimination through aminolysis of metal carboxylate salts. We found that the reaction pathways of the indium precursor, which were critical in terms of controlling the chemical kinetics, in the Masayuki method were more complicated than simple ligand replacement proposed in the literature. We designed a hot-injection approach which separated the ligand replacements of the indium acetate and the aminolysis reactions of the metal precursors. The hot-injection approach was readily applied to the synthesis of ITO nanocrystals with a broad range of tin dopants, leading to products with decent size distributions. Further multiple injections of reagents allowed effective size tuning of the colloidal ITO nanocrystals. We revealed the effective doping of different concentrations of Sn^4+^ ions into the corundum-type lattices of the nanocrystals, resulting in characteristic and tunable near-infrared SPR peaks.

Our study demonstrates that FTIR is a powerful technique for the investigation of the molecular mechanism and precursor conversion pathways associated with the reactions to generate oxide nanocrystals, which may shed light on future rational design of synthetic strategies of oxide nanocrystals.

## Competing interests

The authors declare that they have no competing interests.

## Authors’ contributions

YZJ designed the experiments and wrote the paper. QY preformed most experiments and drafted the figures. YPR and XW carried out some experimental work. ZZY provided a few valuable suggestions. All authors read and approved the final manuscript.

## Authors’ information

YZJ is an associate professor at the Materials Science and Engineering Department of Zhejiang University. ZZY is a full professor at the Materials Science and Engineering Department of Zhejiang University. QY and YPR are master students under the supervision of Dr. Jin. XW is a Ph.D. student co-supervised by Dr. Jin and Prof. Ye.

## Supplementary Material

Additional file 1 ITO nanoflowers (Figure S1), FTIR spectra of the materials (Figure S2), FIR of the ligand replacement reactions (Figure S3), temporal evolution of the morphologies of the ITO nanocrystals (Figure S4), ITO nanocrystals obtained by the Masayuki method (Figure S5), electron diffraction pattern of the ITO nanocrystals (Figure S6), XRD patterns of the tin oxide (Figure S7), and XPS spectra of the ITO nanocrystals (Figure S8). Click here for file
