# Supplementary material for: Molecular mechanism of monodisperse colloidal tin-doped indium oxide nanocrystals by a hot-injection approach
Source: Nanoscale Res Lett. 2013 Apr 2;8(1):153. doi: 10.1186/1556-276X-8-153 (PMC3626570; doi:10.1186/1556-276X-8-153)
Supplement: Additional file 1 — ITO nanoflowers (Figure S1), FTIR spectra of the materials (Figure S2), FIR of the ligand replacement reactions (Figure S3), temporal evolution of the morphologies of the ITO nanocrystals (Figure S4), ITO nanocrystals obtained by the Masayuki method (Figure S5), electron diffraction pattern of the ITO nanocrystals (Figure S6), XRD patterns of the tin oxide (Figure S7), and XPS spectra of the ITO nanocrystals (Figure S8). [file 1556-276X-8-153-S1.pdf]

Supporting Information

Molecular Mechanism of Monodisperse Colloidal Tin Doped Indium Oxide Nanocrystals by a Hot-injection Approach

Yizheng Jin[1,2,3*], Qing Yi[1], Yuping Ren[1], Xin Wang[1], and Zhizhen Ye[1,2*]

[1] State Key Laboratory of Silicon Materials, Department of Materials Science and Engineering, Zhejiang University, Hangzhou 310027, People's Republic of China

[2] Cyrus Tang Center for Sensor Materials and Applications, Zhejiang University Hangzhou 310027, People's Republic of China

[3] College of Chemistry & Materials Engineering, Wenzhou University, Zhejiang Province, Wenzhou 325027, People's Republic of China

Corresponding authors: yizhengjin@zju.edu.cn (Dr. Yizheng Jin) & yezz@zju.edu.cn (Prof. Zhizhen Ye)

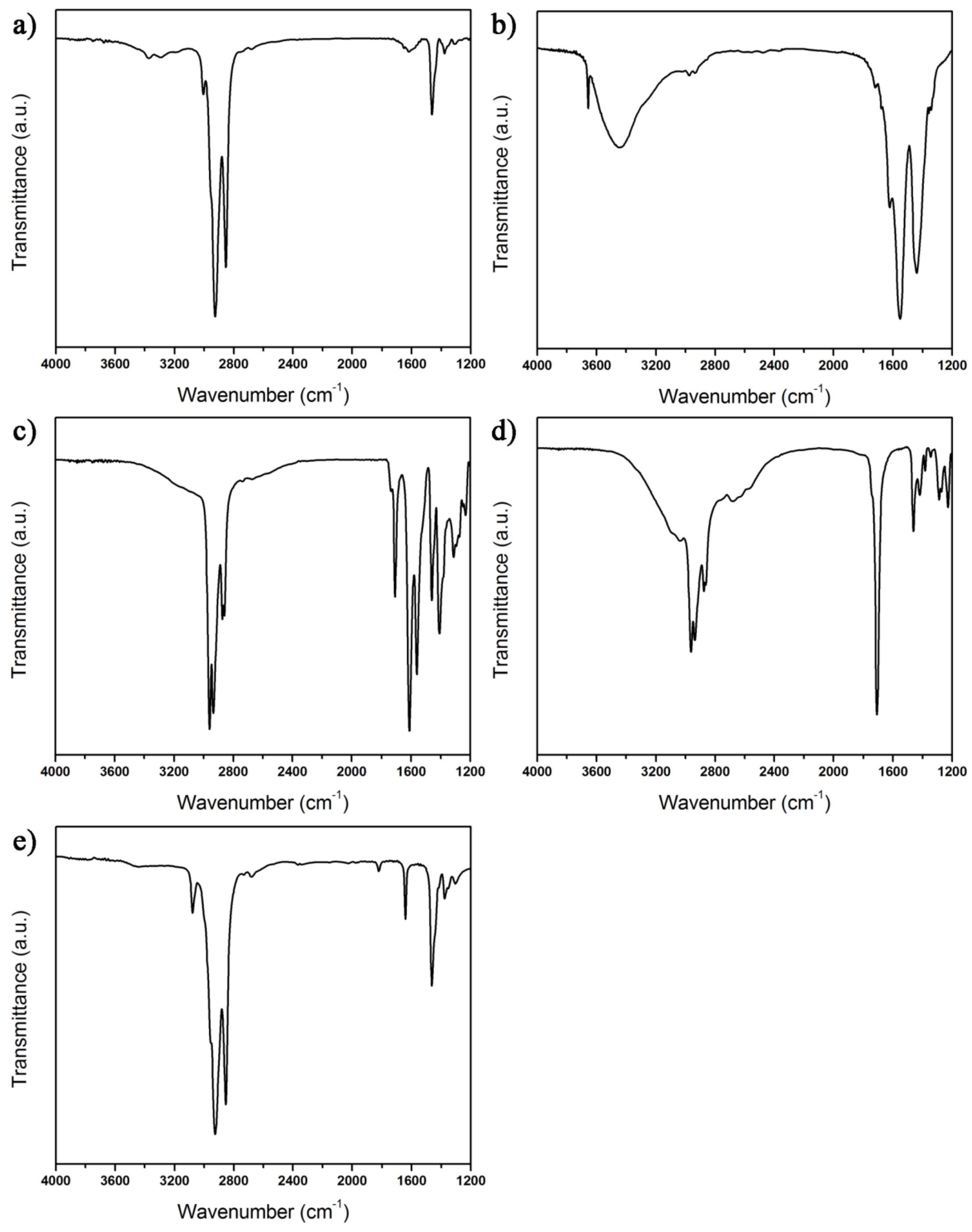

Figure S1 FTIR spectra of a) oleylamine, b) indium acetate, c) tin(II) 2-ethylhexanate, d) 2-ethylhexanatic acid and e) ODE, respectively.

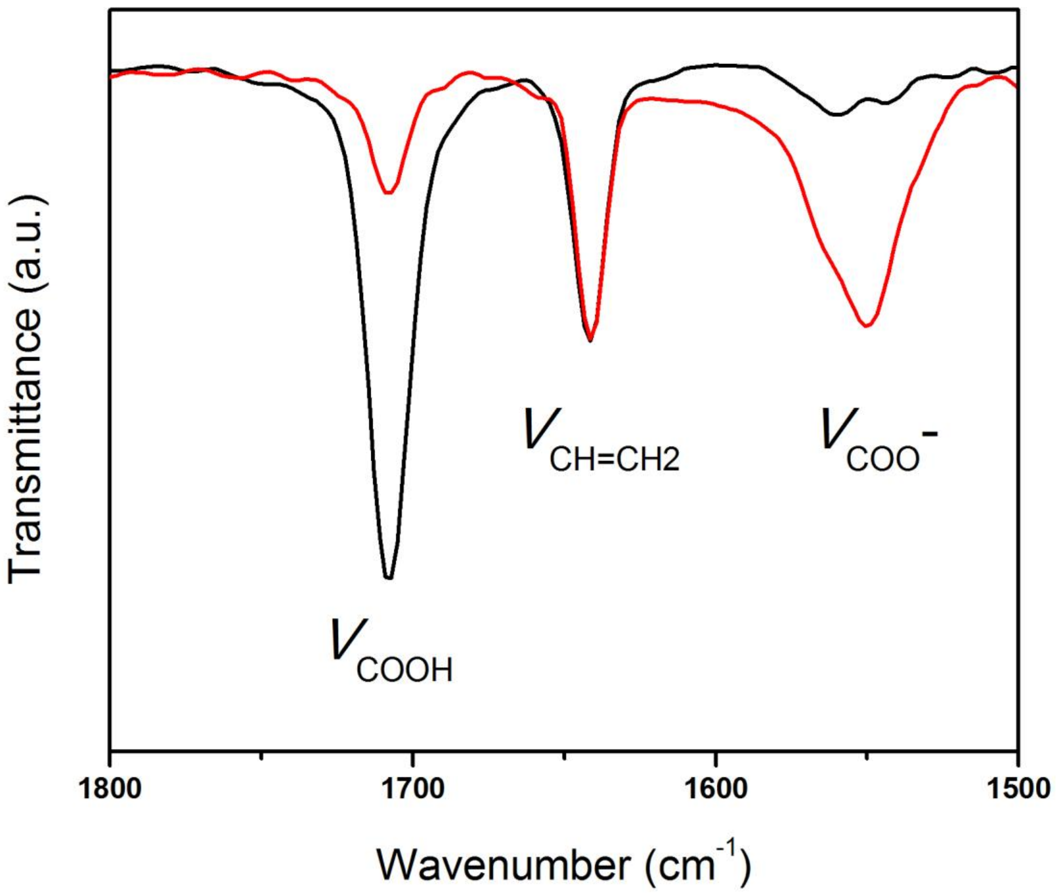

Figure S2 FTIR spectra of the mixture of indium acetate (1.2 mmol), 2-ethylhexanoic acid (3.6 mmol) and ODE (10 ml) before (25 ℃) and after ligand exchange at 290 ℃ for one hour. The proportion of 2-ethylhexanate to the acetate groups was determined by estimating the remaining 2-ethylhexanoic acid in solution derived from the calculations of the absorption peak area in the corresponding FTIR spectra.

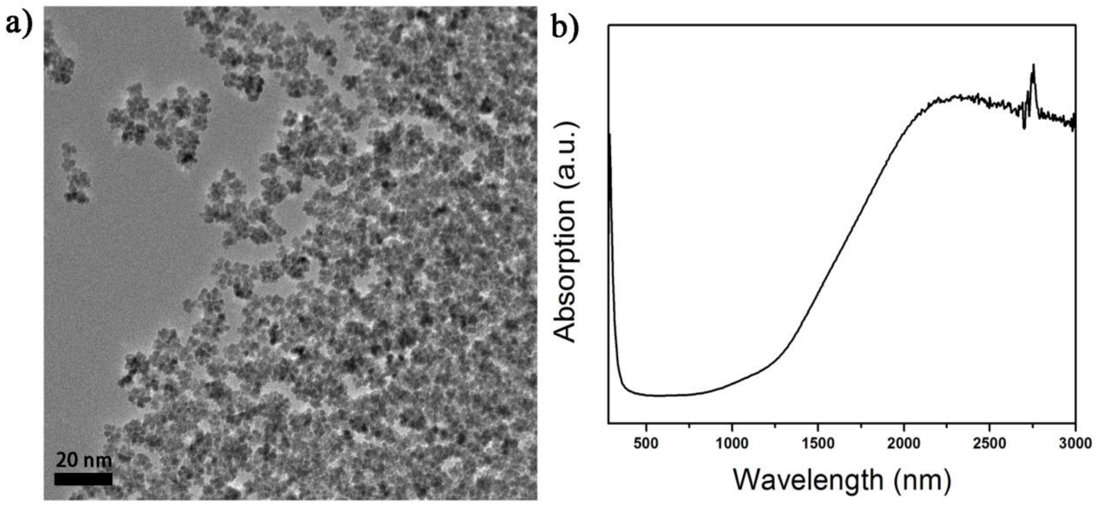

Figure S3 ITO nanoflowers from the reactions using *n*-octanoic acid in the reagents. a) a typical TEM image and b) UV-Vis-NIR spectrum.

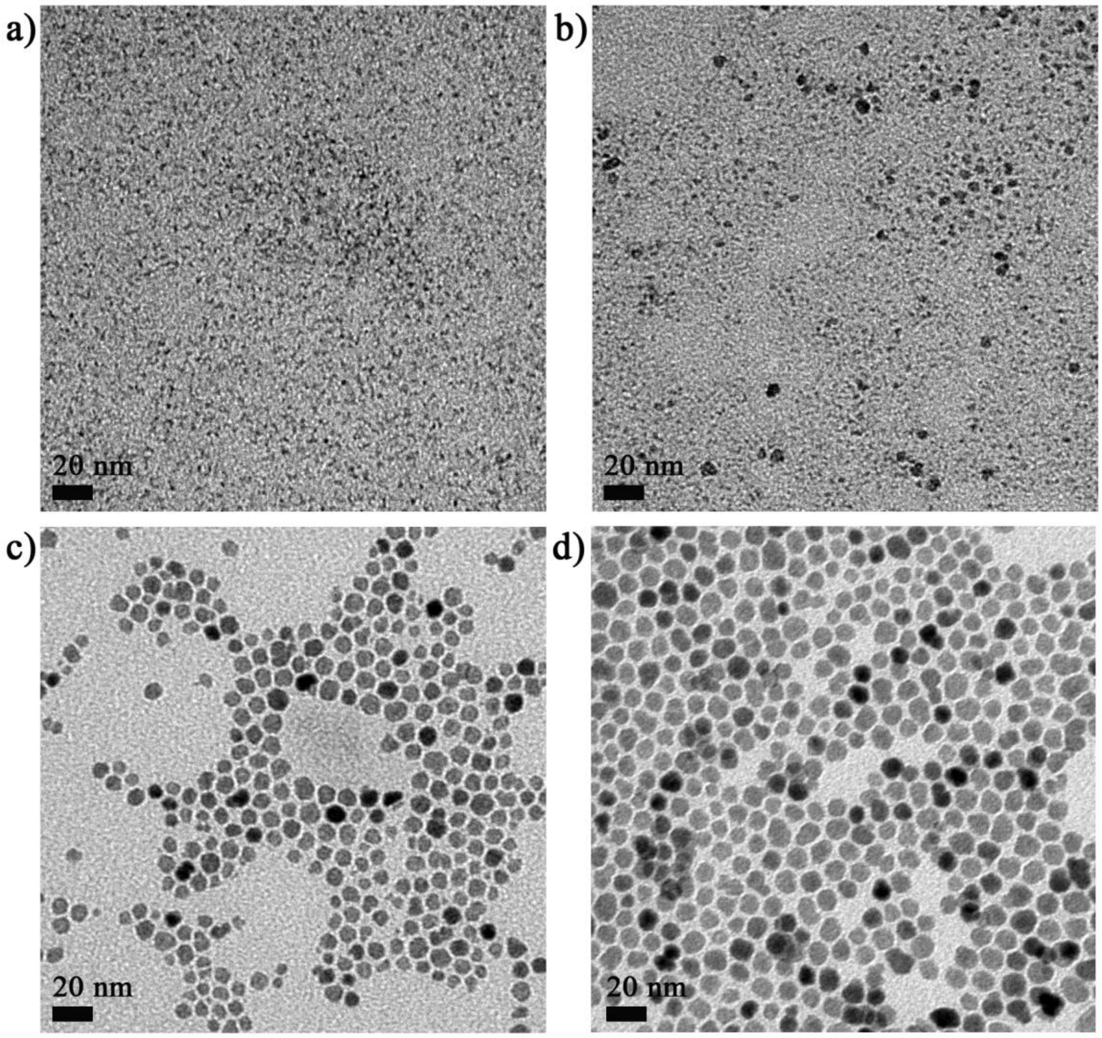

Figure S4 Temporal evolution of the morphologies of the ITO nanocrystals. a)-d)TEM images were recorded from the nanocrystals in the aliquots which were taken from the reaction flask at 3, 8, 20 and 30 min, respectively.

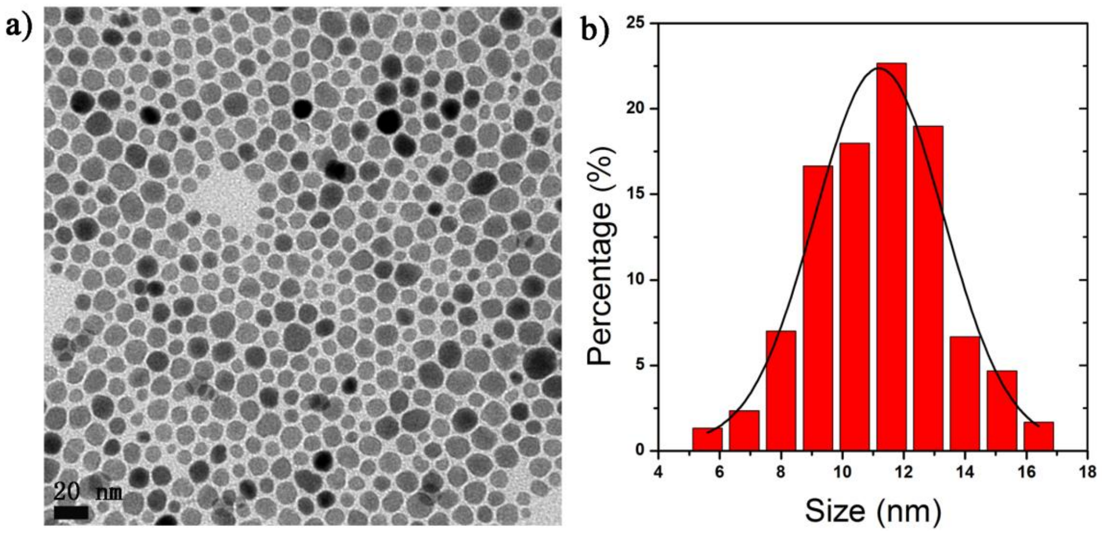

Figure S5 ITO nanocrystals (10 mol.% of tin precursor) obtained by the Masayuki method. a) a typical TEM image and b) the corresponding histogram showing the size distribution of 11.2 ± 2.1 nm.

.

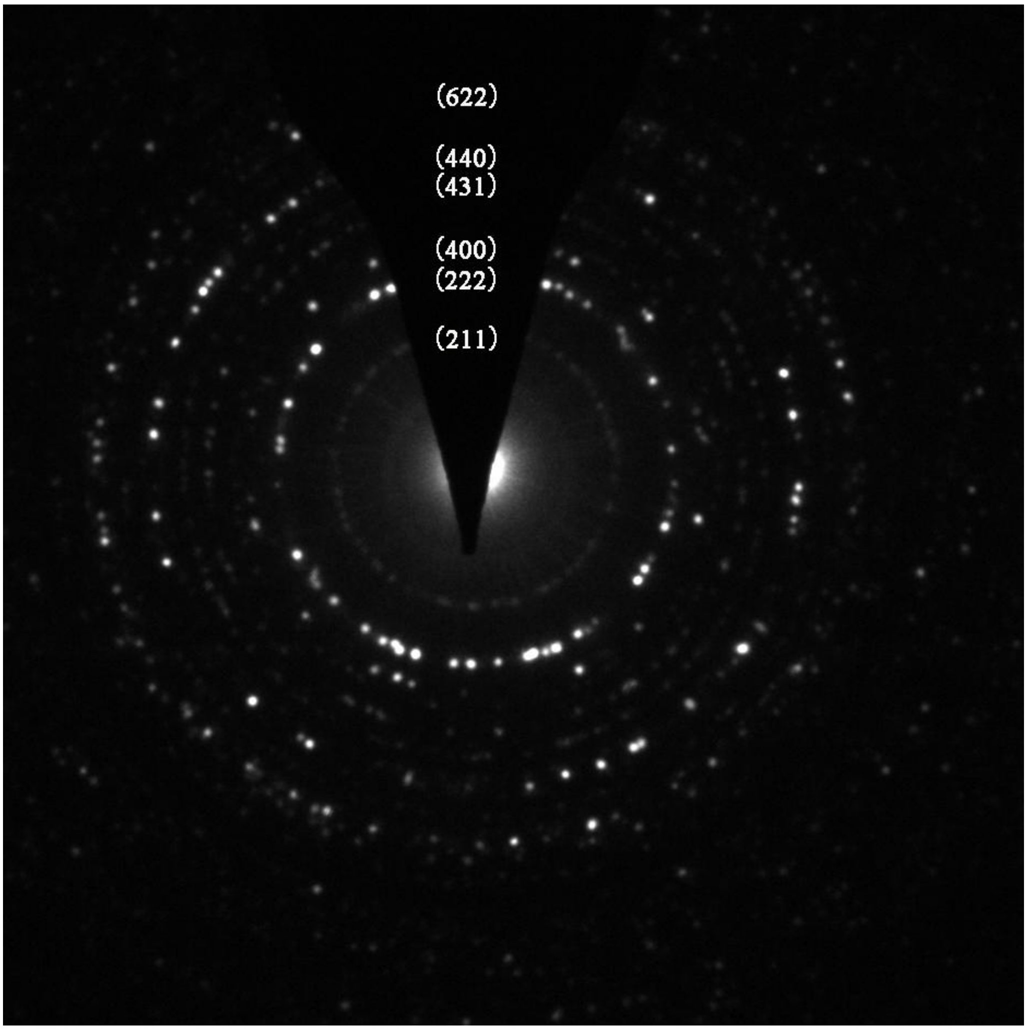

Figure S6 Electron diffraction pattern of the ITO nanocrystals.

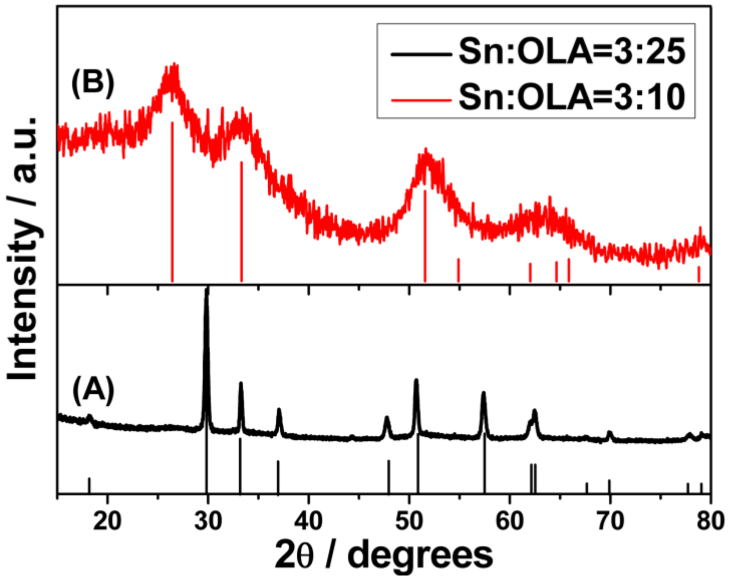

Figure S7 XRD patterns of the products prepared by aminolysis of different amounts of tin(II) 2-ethylhexanoate in oleylamine at 280 ℃ for 1 h. The results suggest that both (A) SnO and (B) SnO_2 (JCPDS patterns of PDF#06-0395 and PDF#41-1445) may be obtained depending on the reaction conditions.

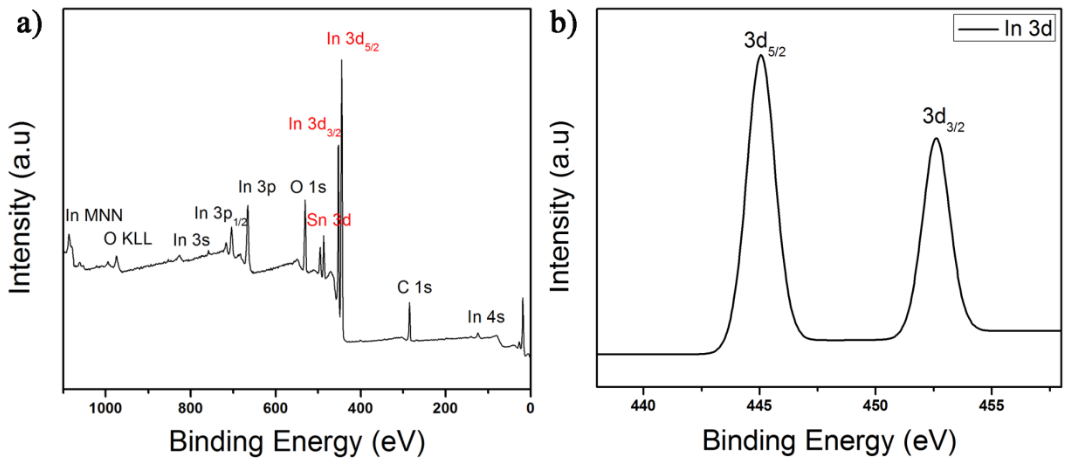

Figure S8 XPS spectra of the ITO nanocrystals from the hot-injection approach.

a) wide scan of the ITO nanocrystals and b) narrow scan of the In 3d peaks.
